# Predicting *Karenia brevis* Induced Respiratory Irritation at Individual Southwest Florida Beaches Using Cell Abundances Plus Wind Direction and Speed

**DOI:** 10.1029/2025GH001664

**Published:** 2026-04-09

**Authors:** K. M. Collins, A. G. Hounshell, B. Kirkpatrick, A. Cook, K. A. Hubbard, M. C. Tomlinson, R. P. Stumpf

**Affiliations:** ^1^ CSS Inc. at National Oceanic and Atmospheric Administration National Centers for Coastal Ocean Science Silver Spring MD USA; ^2^ National Oceanic and Atmospheric Administration National Centers for Coastal Ocean Science Beaufort NC USA; ^3^ Gulf of America Coastal Ocean Observing System Texas A&M University College Station TX USA; ^4^ Mote Marine Laboratory Sarasota FL USA; ^5^ Florida Fish and Wildlife Commission Fish and Wildlife Research Institute St. Petersburg FL USA; ^6^ National Oceanic and Atmospheric Administration National Centers for Coastal Ocean Science Silver Spring MD USA

**Keywords:** *Karenia brevis*, harmful algal bloom, brevetoxins, respiratory irritation, red tide, risk‐level forecast

## Abstract

Nearly annually, blooms of the dinoflagellate *Karenia brevis* form along the southwest Florida coast leading to a variety of negative impacts, including respiratory irritation (RI) in humans. To limit these impacts, NOAA's National Centers for Coastal Ocean Science (NCCOS) developed a RI model to provide beach‐goers with a category‐based estimate of RI risk at individual beaches along Florida's Gulf and Atlantic coasts. The RI model is based on: (a) *K. brevis* cell counts collected at individual beaches; (b) high resolution wind direction and speed forecasts and observations; and (c) point‐based beach shoreline orientation used to designate onshore and offshore winds. To test the model logic, an analysis of modeled RI was compared to same‐day RI reports, based on the frequency of coughs at individual beaches from the Beach Conditions Reporting System (BCRS). Overall, the model proved to be 88% accurate when *K. brevis* was present along the southwest Florida coastline from 2006 to 2022. In addition, validation efforts confirmed model assumptions, including: (a) reports of higher RI correlate with higher *K. brevis* cell abundances; and (b) when cells are present, onshore winds lead to a higher risk of RI. However, individual model categories (“low,” “moderate”) were less robust. Furthermore, BCRS was not a direct measure of toxic aerosol presence, so some coughing (modeled false negatives) may result from other environmental factors. Together, results suggest the RI model accurately predicts “very low” and “high” risk, but that additional research is needed to better capture environmental conditions when RI is “low” or “moderate.”

## Plain Language Summary

Nearly every year red tide (*Karenia brevis*) blooms along the southwest Florida coast lead to a variety of negative impacts, including respiratory irritation (RI) in humans, such as coughing, sneezing, and eye irritation, which is especially harmful to those with underlying respiratory conditions. In order to inform beachgoers of the potential presence and risk‐level of respiratory impacts associated with red tide, NOAA's National Centers for Coastal Ocean Science developed a RI model. The model uses red tide cell abundances collected by regional partners and community scientists coupled with wind speed and direction at individual beaches. In this study, we tested the RI model, using estimates of RI at impacted beaches by trained lifeguards and beach ambassadors through the Beach Reporting Conditions System. Overall, we found that the model was accurate in predicting a very low or high risk of RI due to red tide, but that additional research is needed to better predict instances of low and moderate RI. Specifically, we found that several of the underlying model assumptions were true, including that there was a higher risk of RI with higher red tide cell abundances, especially when winds were blowing onshore.

## Key Points

- Beach‐level *Karenia brevis* cell abundances were positively associated with elevated respiratory irritation (RI) risk at individual beaches
- When *Karenia brevis* cells were present, onshore winds lead to higher risk of RI, as captured by the beach‐level model
- The model accurately captured very low and high risk of RI, but more work is needed to capture low and moderate risk

## 1 Introduction

Harmful algal blooms (HABs) of the toxic dinoflagellate *Karenia brevis*, which are commonly referred to as red tides, produce a suite of toxins, including brevetoxins, which pose multiple health and ecological risks (Fleming et al., 2011; Landsberg et al., 2009). Widespread, negative ecosystem impacts can include fish kills, usually by gill paralysis, as well as manatee and seabird mortalities (Bossart et al., 1998; Fire et al., 2015). In humans, Neurotoxic Shellfish Poisoning can occur when brevetoxins produced by *K. brevis* accumulate in consumed shellfish (Watkins et al., 2008). Additionally, brevetoxins produced by *K. brevis* can become aerosolized due to wave and wind action, posing a significant human health risk along beaches and in areas where recreation occurs (Kirkpatrick et al., 2004; Pierce, 1986; Pierce et al., 2005). People with underlying chronic respiratory conditions, such as asthma, may experience particularly severe symptoms, which can lead to increased emergency room visits in red tide impacted areas (Kirkpatrick et al., 2006). Even in people without underlying respiratory conditions, the aerosolized toxins can cause respiratory irritation (RI) with a variety of symptoms, including coughing, running nose, and eye irritation (Backer et al., 2003). As a result, people may avoid the beach and beachside businesses when red tide is present, resulting in decreased revenue leading to billions of dollars in annual economic losses during a red tide event (Alvarez et al., 2024; Ferreira et al., 2022; Larkin & Adams, 2007).

Multiple methods are used to limit and mitigate the ecosystem, human health, and economic impacts of red tide on Florida Gulf coast communities, including robust strategies for *K. brevis* monitoring (FWRI, 2024; Kirkpatrick et al., 2008), the development of early warning and forecasting systems (Stumpf et al., 2003, 2009), and a variety of response and clean‐up efforts by non‐profit, state, and county agencies (Hoagland et al., 2020). For example, the Florida Fish and Wildlife Conservation Commission Fish and Wildlife Research Institute (FWC‐FWRI) maintains a routine *K. brevis* monitoring program which collects samples for microscopic identification and enumeration of *K. brevis* cells at fixed sites at regular intervals, along with a robust event response program when a bloom is observed (FWRI, 2024). In addition, county governments (e.g., Pinellas County) and other Florida research and non‐profit institutions (e.g., Mote Marine Laboratory, Sanibel Captiva Conservation Foundation (SCCF), among others) also have routine and event‐driven *K. brevis* microscopic monitoring programs that further supplement state efforts (FWRI, 2024). These *K. brevis* monitoring efforts provide vital information on the current location and intensity of red tide along the Florida coastline, particularly in southwest Florida, which can help avoid human exposure and limit negative impacts from red tide.

Early‐warning and forecasting systems can provide an additional advance warning of bloom movement and impacts. In 2001, NOAA began a *K. brevis* satellite monitoring and forecasting system to supplement existing in situ monitoring efforts (Stumpf et al., 2003). Originally, 3‐day forecasts of potential RI risk (e.g., Very Low, Low, Moderate, or High risk of RI) were issued at the county level 1–2 times per week based on bloom location and near‐term weather conditions. An analysis of this initial forecast found that it was 90% accurate at the county level, meaning if RI was forecasted in the county over the next few days that RI was observed somewhere in the county during the forecasted time period (Stumpf et al., 2009). However, when evaluated at the individual beach level, the RI forecast was only 22% accurate. The analysis suggested there was insufficient spatial and temporal resolution to accurately predict beach‐level impacts with the county‐level forecast structure. Since the original RI forecast, *K. brevis* in situ monitoring and wind forecast models have seen significant improvements in spatial and temporal resolution, allowing for the development of more accurate beach‐level *K. brevis* RI forecasts.

A collaboration between NOAA and the Gulf of America Coastal Ocean Observing System (GCOOS) further expanded in situ *K. brevis* monitoring via the HABscope network (GCOOS, 2024; Hardison et al., 2019). Briefly, the HABscope network leverages community scientists located across the Florida Gulf coast to augment routine state and county supported *K. brevis* monitoring efforts. The HABscope network uses a low‐cost microscope combined with a cell phone to capture videos of collected water samples for *K. brevis* cells. Artificial intelligence algorithms are then used to identify and quantify *K. brevis* cells in near‐real time based on their morphology and swimming behavior in the collected videos (GCOOS, 2024; Hardison et al., 2019). In 2022 alone, an additional 86 beach locations were sampled by the HABscope network for an additional 3,127K*. brevis* cell counts. The integration of higher temporal and spatial resolution wind forecasts with expanded sampling efforts allowed for significant enhancements to the NOAA RI forecast in 2019. As compared to previous county‐level forecast efforts, the updated RI forecast provides beach‐level forecasts of RI risk at three‐hour intervals over the subsequent 30 hr, whenever a *K. brevis* cell abundance sample is available.

While *K. brevis* monitoring and forecasting efforts seek to provide guidance and information on current conditions and short‐term impacts, additional efforts to respond to the impacts of red tide are necessary to reduce negative impacts of *K. brevis* on coastal communities. To quantify the RI impacts of *K. brevis*, Mote Marine Laboratory implemented the Beach Conditions Reporting System (BCRS) to provide information on beach‐level impacts of red tide. Starting in 2006, BCRS developed a network of trained lifeguards and beach ambassadors who “listen” to beachgoers for the presence and/or frequency of coughing (Kirkpatrick & Currier, 2010; Kirkpatrick et al., 2008). The presence and/or frequency of coughing is then categorized (None, Slight, Moderate, or Intense RI) and used as a proxy for RI (e.g., coughing, sneezing, and throat and eye irritation; Backer et al., 2003; Fleming et al., 2005, 2007). In addition, BCRS includes reports of current surf conditions, water clarity/color, presence of dead fish, and approximate wind direction and speed, which can be used to further support the occurrence and impacts of red tide. Importantly, the standardized, beach‐level RI reporting by BCRS provides a robust validation data set for the southwest Florida RI mode.

In this study, we validated the beach‐level RI model that underlies the updated NOAA RI forecast. We used a combination of *K. brevis* cell abundance data collected by state and local partners and the HABscope network, combined with observed wind speed and direction data from the NOAA National Data Buoy Center (NDBC), to model RI at individual southwest Florida beach locations from 2006 to 2022. Modeled RI risk (as Very Low, Low, Moderate, or High) was compared to BCRS beach‐level RI reports (None, Slight, Moderate, or Intense). Through this validation effort, we explored the three underlying assumptions of the beach‐level RI model: (a) Higher observed *K. brevis* cell abundances lead to a higher risk of RI; (b) There is a lower risk of RI with offshore winds; and (c) Higher onshore wind speeds lead to increased risk of RI.

## 2 Methods

### 2.1 Respiratory Irritation Model

**Figure 1:**
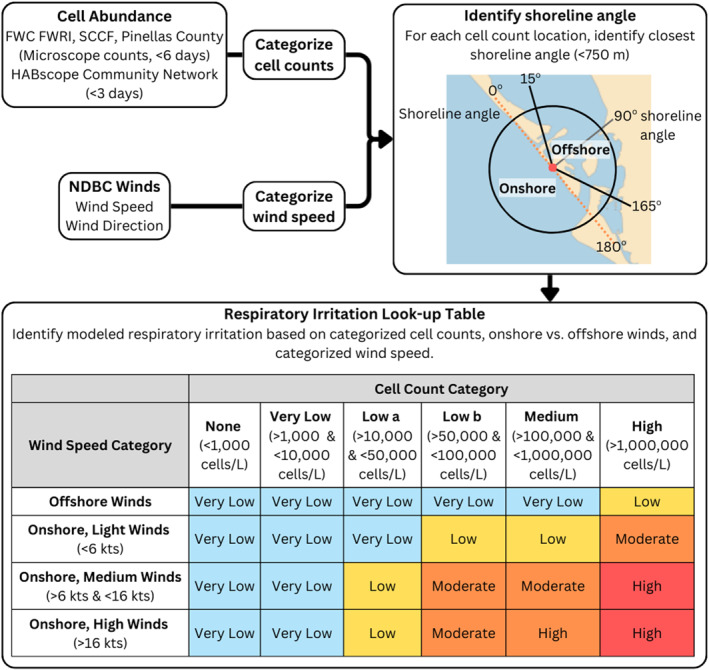
Conceptual diagram of the respiratory irritation (RI) forecast model. The model used *K. brevis* cell abundance data collected by state sampling and the HABscope network plus observed or forecasted wind speed and direction. Cell abundances were passed through an age filter and categorized into cell abundance categories. Wind speed was categorized as onshore or offshore, based on the beach shoreline angle, and then further classified as light, medium, or high winds. Modeled RI was then assigned based on cell abundance category, on‐ versus offshore winds, and wind speed.

The RI model uses a combination of *K. brevis* cell abundances collected at individual beaches along the southwest Florida coast plus observed or forecasted wind speed and direction obtained from NOAA's NDBC for hindcasts and NOAA's National Digital Forecast Database (NDFD; Figure 1) when in forecast mode. The model is designed to provide an estimate of RI risk at individual beaches to alert beach‐goers, public health officials, and natural resource managers with information about potential risks associated with *K. brevis* blooms along the Florida coastline, primarily along the southwest Florida Gulf coast. *K. brevis* cell abundances were obtained from several Florida partners, including FWC FWRI and the HABscope community network. Prior to use in the RI model, cell abundances were filtered by age, where microscope counts (e.g., from FWC FWRI) greater than 6 days old and HABscope cell abundances greater than 3 days old were removed.

Remaining cell observations were then categorized by cell abundance category (None/Background, Very Low, Low a, Low b, Medium, and High; Figure 1). Similarly, NDFD winds were first categorized as onshore versus offshore winds, with onshore winds further categorized by wind speed (Light, Medium, and High). Based on the cell abundance and wind categories, a modeled risk of RI was generated based on a lookup‐table (Figure 1). To assess the underlying assumptions and performance of the model, we generated hindcasts of RI risk at individual beaches along the southwest Florida coast from 2006 to 2022. The modeled risk was compared to RI as measured by Mote Marine Laboratory's BCRS at individual beach locations (Kirkpatrick et al., 2008).

### 2.2 Data Collection

#### 2.2.1 *Karenia brevis* Cell Abundance

**Figure 2:**
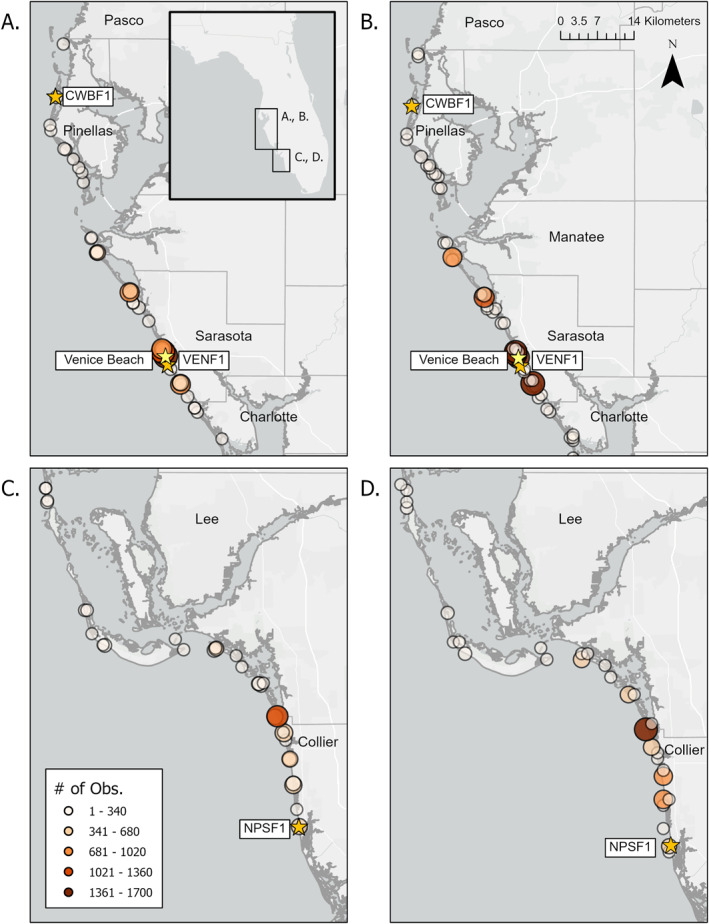
Study area (inset in A) and location sites showing number of Beach Conditions Reporting System observations from (a) Pinellas to Charlotte County and (c) Lee to Collier County for the period from 2006 to 2022. The frequency of *K. brevis* cell abundance observations for the same period are shown for (b) Pinellas to Lee County and (d) Lee to Collier County. National Data Buoy Center buoy locations (CWBF1, VENF1, NPSF1) are marked with orange stars, while the yellow star notes the location of Venice Beach.

We combined state and local sources of *K. brevis* cell abundance data, including from FWC FWRI, Pinellas County, SCCF, and the HABscope community volunteer network (Figure 2). Surface water samples from FWC FWRI, Pinellas County, and SCCF were collected using each program's water sampling methods and preserved with un‐acidified Lugol's solution. Light microscopy enumeration across groups followed the same general methods. Preserved cells were sub‐sampled by settling in 0.5–3 mL aliquots into Nunc^®^ chambers prior to enumeration via light microscopy (e.g., Hu et al., 2022). Cell abundance data was obtained directly from regional partners or via the open‐data platform, HABSOS (NOAA NCEI, 2014), and converted into the cell abundance bins (Figure 1). Cell abundances from the HABscope community network were collected by volunteers along the Florida Gulf Coast who routinely monitor beaches and other locations for *K. brevis* cell abundances. GCOOS, in collaboration with NOAA's National Centers for Coastal Ocean Science (NCCOS), maintains the network. HABscope uses a student microscope paired with an iPOD to capture 30 s videos of collected water samples. Collected videos were uploaded to an online repository where AI paired with human screening was used to identify and count *K. brevis* cells based on their cell morphology and occurrence of movement (see Hardison et al., 2019 for more details). For model validation, we constrained cell abundance data obtained from FWC FWRI partners and the HABscope community network to those that were paired with a same‐day BCRS respiratory report (see below) collected from 2006 to 2022 for the respiratory model assessment.

#### 2.2.2 Wind Direction and Speed

Hourly observed wind direction and speed data for the study period was obtained from NOAA's NDBC (ndbc.noaa.gov) for stations along the coast of southwest Florida, including at Clearwater (CWBF1), Venice (VENF1), and Naples (NPSF1; Figure 2). Observed cell abundances were then matched to the nearest hourly observation of wind speed and direction from the closest buoy location (Figure 2).

#### 2.2.3 Beach Conditions Reporting System (BCRS)

We used the BCRS program to validate the RI model output (Figure 2). BCRS categorizes the number of coughs observed at individual beaches by trained BCRS observers and lifeguards into None, Slight, Moderate, and Intense RI (Kirkpatrick & Currier, 2010; Kirkpatrick et al., 2008). None corresponds to no coughing observed; Slight corresponds to coughing/sneezing observed every 30 s; Moderate corresponds to coughing/sneezing observed every 5 s; and Intense corresponds to coughing/sneezing heard continuously. While not a direct measure of RI due to *K. brevis* and associated brevetoxins, the database is routinely used as a proxy for RI due to *K. brevis* exposure at southwest Florida beaches (Kirkpatrick et al., 2008).

#### 2.2.4 Shoreline Orientation

For each beach sample location, we identified the shoreline orientation using the Florida Coastal Range Monuments database (FDEP, 2017) which includes shoreline orientation data for individual points along the southwest Florida coast between 200 and 500 m apart. For each beach sample location, we then assigned a 90° shoreline angle based on the shoreline orientation identified above, which was used to designate offshore versus onshore winds (Figure 1). Offshore winds correspond to 90° ± 75° (15°–165°) of the shoreline angle, meaning that winds nearly parallel (<15° and 165°–180°) to the shore were considered onshore. This was a conservative estimate of onshore winds, which allows for slight discrepancies in wind direction between the wind measurement site and the beach location which could lead to actual occurrence of aerosols at the beach location.

### 2.3 Model Assessment and Validation

#### 2.3.1 Confirming *K. brevis* Presence Along the Southwest Florida Coast

**Figure 3:**
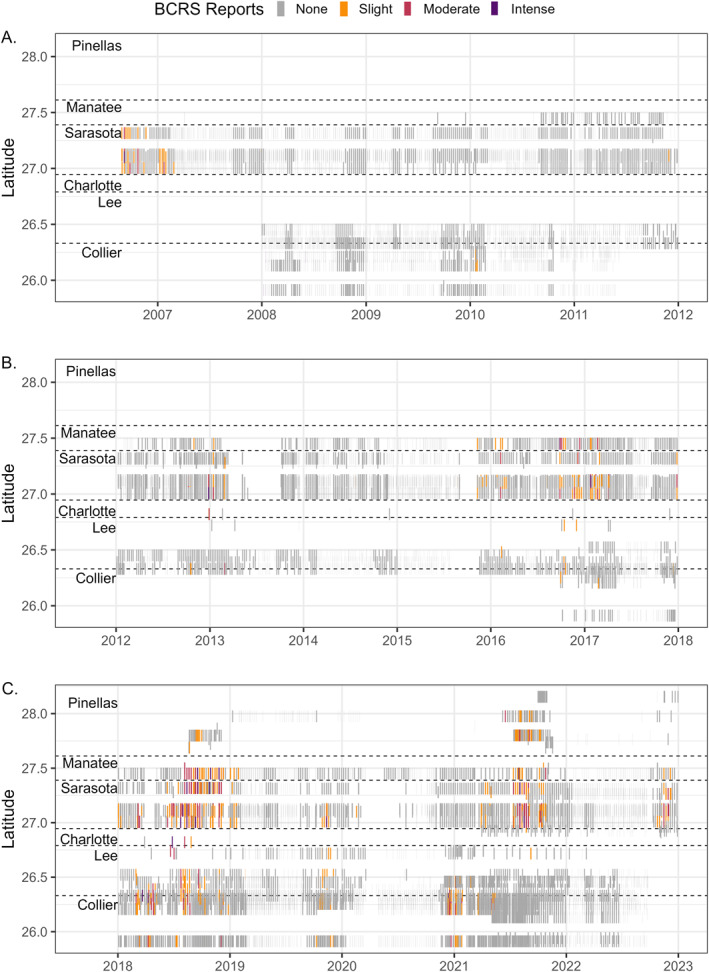
Matched Beach Conditions Reporting System (BCRS) reports and observed *K. brevis* cell abundances collected along the southwest Florida coast from Pinellas to Collier Counties which were used for model validation from (a) 2006–2011, (b) 2012–2017, and (c) 2018–2022. Colors correspond to the respiratory irritation intensity observed by BCRS from none (gray) to intense (purple). Light gray bars correspond to time periods when *K. brevis* was not observed along the southwest Florida coast and which were omitted from the model validation. Horizontal dashed lines separate Florida counties, from Pinellas to Collier County.

The overall goal of the model was to predict periods of RI risk at individual beach locations during a red tide event (e.g., when *K. brevis* cells were present and wind conditions were conducive to onshore aerosolization and transport; Pierce et al., 2005). *K. brevis* typically occurs seasonally, from about August to February, though blooms can persist into the spring and summer depending on environmental conditions (Stumpf et al., 2009, 2022). To ensure we were appropriately assessing the accuracy of the model when *K. brevis* was geographically present, we constrained the model validation period to only times when *K. brevis* was detected along the southwest Florida coast. We assumed *K. brevis* was geographically present if the hindcasted RI occurred within ±15 days and ±0.5° latitude of any additional and/or confirmed detection of *K. brevis* cells above background concentrations (*K. brevis* > 1,000 cells L^−1^). Modeled hindcasts of RI that did not meet this criterion were removed from subsequent analyses. This ensured model validation was focused on periods when there was a reasonable possibility of RI due to red tide and excluded extended periods when the model would be “correct” only because *K. brevis* was not present/observed anywhere along the southwest Florida coast (Figure 3).

#### 2.3.2 Model Validation

We applied the RI model for data from 2006 to 2022 using observed *K. brevis* cell abundances, wind speed and direction, and shoreline orientation to estimate the risk of RI at individual beaches from Pinellas to Collier counties during conditions when *K. brevis* was present along the southwest Florida coast (as defined in 2.3.1). We then matched the remaining RI model output (categorized as Very Low, Low, Moderate, and High RI risk) to same‐day BCRS reports of RI (categorized as None, Slight, Moderate, and Intense RI, respectively) based on a strict temporal (±0.007° ≈750 m) and spatial (±60 min) filter for matched modeled hindcasts to observation validation.

We tested the underlying assumptions of the RI model, including that: (a) there was a higher risk of RI with higher observed *K. brevis* cell abundances; (b) there was a higher risk of RI with onshore winds; and (c) higher, onshore wind speeds led to increased risk of RI. We used all matched cell abundance‐BCRS RI reports when *K. brevis* was present along the southwest Florida coast to test the first assumption, relating RI with cell count concentrations. For the second assumption about wind direction, we constrained the analysis to a single location with the highest number of matched cell count‐BCRS reports (Venice Beach, Florida; *n* = 956).

We also calculated a series of statistical metrics to test the accuracy of the model using a confusion matrix between modeled and observed BCRS RI (Table 1). A confusion matrix identifies true positives (TP) and true negatives (TN) as well as false positives (FP) and false negatives (FN) between the modeled and observed RI (e.g., Congalton, 1991). A TP refers to an exact match between the categorical forecast and the categorical observation while a TN corresponds to instances when the categorical forecast does not match the categorical observation. A FP refers to an instance where the model overpredicts the categorical condition (e.g., modeled = High, observed = None) and an FN corresponds to an instance where the model underpredicts the categorical condition (e.g., modeled = Very Low, observed = Intense). Statistical metrics analyzed included user's accuracy (probability of detection), success ratio, bias, and equitable threat score (Gilbert skill score), as defined in Table 2. User's accuracy captures whether the RI categories were correctly identified while bias identifies potential model over‐ or under‐estimation. The success ratio captures how often the model matches RI observations while the equitable threat score considers how well the model performs relative to chance.

**Table 1:** Multi‐Category Contingency Table Showing Instances of Matched Modeled and Observed Respiratory Irritation From 2006 to 2022

	Observed respiratory irritation (BCRS)					
		None	Slight	Moderate	Intense	Total
Modeled Respiratory Irritation	Very Low	8,087	530	111	9	8,737
	Low	294	171	104	14	583
	Moderate	145	75	31	5	256
	High	66	104	83	43	296
	Total	8,592	880	329	71	9,872

*Note.* True positives for each category are highlighted in gray. Values below the gray boxes correspond to false positives while values above the gray boxes correspond to false negatives.

**Table 2:** Selected Model Validation Statistics Used to Assess the Modeled Respiratory Irritation (RI) as Compared to Observations (Beach Conditions Reporting System Observed RI) for Very Low, Low, Moderate, and High Modeled RI

	Description	Equation	Perfect score	Modeled respiratory irritation			
				Very low	Low	Moderate	High
Prevalence	How often does the RI condition actually occur?	(TP + FN)/Total	NA	87%	9%	3%	1%
User's Accuracy (Probability of Detection)	What fraction of the observed RI categories were correctly modeled?	TP/(TP + FN)	1.0	0.94	0.19	0.09	0.61
Success Ratio	What fraction of the modeled RI events were correctly observed?	TP/(TP + FP)	1.0	0.93	0.29	0.12	0.15
Bias	If less than 1 under‐modeling, if greater than 1 over‐modeling	(TP + FP)/(TP + FN)	1.0	1.02	0.66	0.78	4.17
Equitable Threat Score (Gilbert Skill Score)	Measures observed and/or modeled correctly, adjusting for TPs due to random chance	See Gilbert (1884), Schaefer (1990)	1.0	0.29	0.10	0.04	0.13

*Note.* TP = True Positive; FN = False Negative; and FP = False Positive.

The RI model hindcasts and all statistical analyses were conducted in Python (ver. 3.12.9; Python Software Foundation, 2023). All graphs were generated in R using the ggplot2 package (ver. 4.4.1; R Core Team, 2024). Model code associated with the modeled RI and associated analysis are available via Zenodo (https://doi.org/10.5281/zenodo.17965858).

## 3 Results

### 3.1 Model Validation Data Set

There were a total of 17,026 matched *K. brevis* cell abundances and BCRS RI observations from 2006 to 2022 (Figure 3). Of these time points, 11,631 (68%) occurred when *K. brevis* was present along the southwest Florida coast and thus were retained for analysis and model validation (Figure 3). Importantly, this study period captured multiple significant *K. brevis* blooms along the southwest Florida Coast, including in 2006, 2018, and 2021, along with some smaller, more geographically constrained bloom events in 2013, 2016, 2017, 2019, and 2022 (Figure 3; Stumpf et al., 2022). The majority of matched reports occurred in Sarasota County, where the BCRS was first instituted in 2006 (Kirkpatrick et al., 2008). In 2008, the program expanded to other Florida counties, primarily Lee and Collier Counties, before further expanding to Pinellas County in 2012 (Figure 3).

### 3.2 Cell Abundances

The RI model assumes that there is a greater risk of RI when higher *K. brevis* cell abundances are observed. Overall, we found there was a strong association between *K. brevis* cell abundances and reported BCRS RI, with a higher intensity of RI observed with higher *K. brevis* cell abundances. However, a substantial proportion of samples with confirmed *K. brevis* cell abundances were not associated with any RI (2,244 reports, 19%; Table S1 in Supporting Information S1). For an even smaller proportion of samples, *K. brevis* cell abundances were not above our likely RI threshold (*K. brevis*<1,000 cells L^−1^) yet a range of RI was reported from slight to intense (212 reports, 2%; Table S1 in Supporting Information S1).

### 3.3 Wind Direction

**Figure 4:**
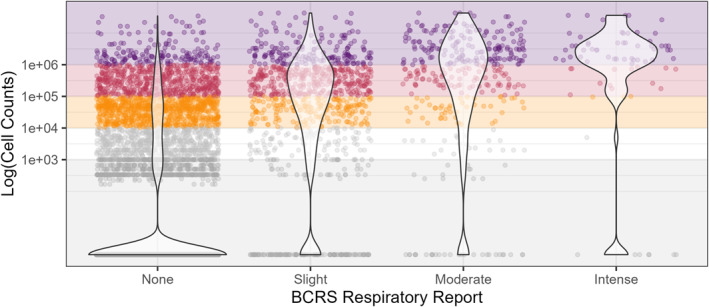
Beach Conditions Reporting System respiratory irritation reports plotted against same‐day collected *K. brevis* cell abundances at individual, matched beach locations along the southwest Florida coast during the study period, when *K. brevis* was geographically present (2006–2022; *n* = 11,631 matched observations).

**Figure 5:**
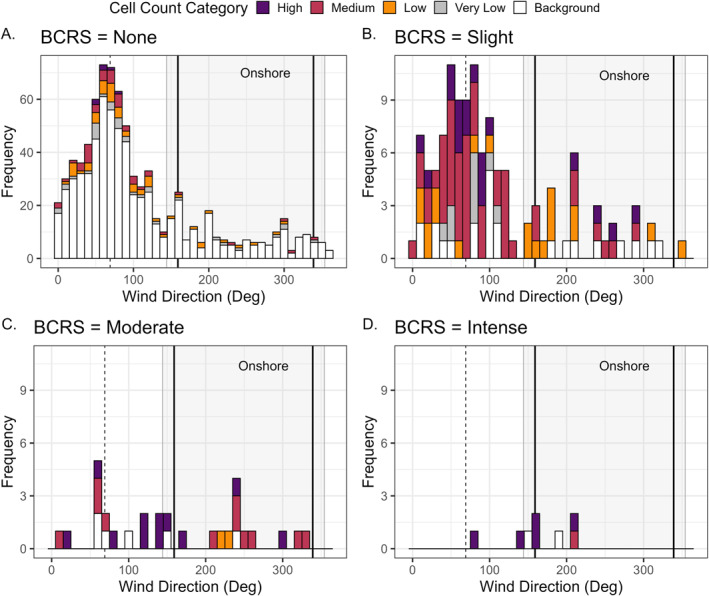
Frequency plots of observed wind direction (degrees) of the Beach Conditions Reporting System (BCRS) respiratory irritation (RI) categories: (a) None, (b) Slight, (c) Moderate, and (d) Intense for Venice Beach, Florida from 2006 to 2022 (*n* = 956). Colors correspond to the observed *K. brevis* cell abundance category at the time of the BCRS RI report. Dashed, vertical lines correspond to the 90° shoreline angle (69°). Solid, vertical lines indicate the 90° shoreline angle ± 90° (159° and 339°, respectively). The light gray box indicates the wind directions corresponding to onshore winds (shoreline angle between 144° and 354° following Figure 1). Note the change in *y*‐axis scale for BCRS = None.

To facilitate the analysis of wind direction on observed RI, we focused our analysis on Venice Beach, Florida (*n* = 956 matched reports). Overall, offshore winds were more common than onshore winds (Figure S1 in Supporting Information S1); however, the distribution of winds for different levels of RI was noticeably different, primarily for moderate and intense RI (Figure 5). In addition, the majority of observed BCRS RI were categorized as none (*n* = 797) followed by slight (*n* = 121) and substantially less reports of moderate (*n* = 30) or intense (*n* = 8) RI at this location which follows similar trends observed across the southwest Florida coast (Figure 4). Offshore winds were more common than onshore winds during BCRS reports of none and slight RI compared to reports of moderate and intense RI, where the offshore and onshore winds were about equal in frequency. While the likelihood and severity of BCRS‐reported RI increased with increasing cell abundances (Figure 4), there were some modulations, likely due to variations in wind direction (Figure 5). During the analysis period, the majority of BCRS observations which reported no risk of RI (e.g., none) occurred when *K. brevis* cell abundances were at none/background levels, regardless of on‐ or off‐shore winds (*n* = 657, 69%; Figure 5). For BCRS observations reporting no risk of RI when cells were present, the majority of instances occurred during offshore winds (*n* = 115; 82% of BCR = none and *K. brevis* abundance>1,000 cells L^−1^; Figure 5). When RI was reported (BCRS ≥ slight), the majority of reports coincided with the presence of *K. brevis* at individual beaches (*n* = 128, 81%; Figure 5). Slight RI was proportionately similar for offshore winds and onshore winds. Most BCRS‐reported RI occurred when winds were offshore, although these were dominated by slight RI (*n* = 107, 67%; Figure 5), regardless of cell abundance category.

### 3.4 Wind Speed

**Figure 6:**
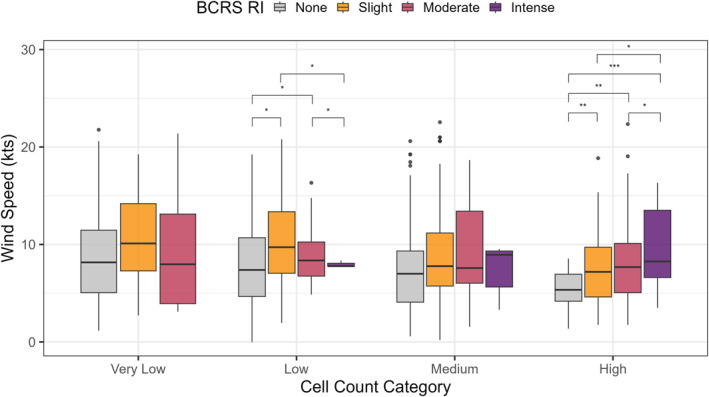
Boxplots showing the distributions of wind speed when cell abundances were present (*K. brevis* > 1,000 cells L^−1^) and winds were onshore for cell abundance categories grouped by Beach Conditions Reporting System respiratory irritation report. Boxes represent the 25th and 75th percentile, respectively, and the thick line shows the median wind speed (knots, kts). Points represent outliers. Statistical significance among groups was tested using Kruskal Wallis with post‐hoc Cliff's delta where: * = small, ** = medium, and *** = large effect sizes (Table S2 in Supporting Information S1; Meissel & Yao, 2024).

The RI model assumes that if *K. brevis* cells were present and winds were onshore, risk of RI increases with increasing wind speed. To test this hypothesis, we selected instances when winds were onshore and cells were present (cell abundances > 1,000 cells L^−1^) across all locations (*n* = 1,076, 11% of all matched reports; Figure 6). At high cell abundances (>1,000,000 cells L^−1^), RI intensity increased from none to intense with increasing wind speed, from a median of 5.4 ± 0.3–8.3 ± 0.6 kts, respectively. In addition, there were significant differences between median wind speed for BCRS reports of none (7.4 ± 0.3 kts) and slight (9.4 ± 0.5 kts) RI at low cell counts (Figure 6).

### 3.5 Respiratory Irritation Model Validation

We conducted several statistical analyses to assess model accuracy using matched observed and modeled RI for individual beaches from 2006 to 2022 (Tables 1 and 2). Even during periods when *K. brevis* was geographically present (±15 days; ±0.5° Lat), the majority of both modeled and observed RI were very low/none (87%), with a total of 8,807 TPs, and decreasing instances of low/slight, moderate, and only 1% occurrence of high/intense RI for both modeled and observed RI (Tables 1 and 2). Along with having the highest occurrence, the very low/none category also had the highest user's accuracy (0.94), success ratio (0.93), and the least amount of bias (1.02), but a relatively low equitable threat score (0.30; Table 2). The low/slight, moderate, and high/intense RI categories had a range of user's accuracies from 0.09 for the moderate category to 0.61 for the high/intense category. The moderate category had a reasonable bias (0.78), while the high/intense category's high bias (4.17; Table 2) indicated that it substantially over‐modeled instances of high/intense RI.

## 4 Discussion

Overall, the RI model demonstrated strong skill and proved to be 88% accurate (TP + TN/Total Predictions) when *K. brevis* blooms were geographically present along the southwest Florida coast from 2006 to 2022. However, some modeled RI categories (e.g., low and moderate), were much less accurate (19% and 9%, respectively; Table 2). Here, we discuss the three underlying assumptions of the RI model and how they relate to BCRS RI observations at southwest Florida beaches, including that: (a) there is a higher risk of RI with higher *K. brevis* cell abundances; (b) there is a much lower risk of RI with offshore winds; and (c) higher wind speeds lead to increased risk of RI. We also discuss possible improvements to the model and model validation methods, including the impact of inherent assumptions of the RI model as well as in the BCRS validation data set.

### 4.1 Cell Abundance as the Primary Indicator of RI Risk Due To Red Tide

Overall, we found a strong link between observed *K. brevis* cell abundance and BCRS reported RI (Figure 4); however, there was still substantial variability in the data. Previous research has shown widespread RI is observed when *K. brevis* cell abundances are >100,000 cells L^−1^ (medium category), with higher RI observed during onshore winds (Kirkpatrick et al., 2008; Stumpf et al., 2003, 2009). Thus, we might expect intense RI reports during medium/high cell abundances, slight or moderate RI reports during low abundances, and none or slight RI reports during background/very low cell abundances. In our analysis, intense RI occurred almost exclusively with cell abundances >100,000 cells L^−1^ (medium or high cell abundances; Figure 4). In addition, with increasing reported RI (slight, moderate), there was a decreasing proportion of associated background/very low cell abundances (Figure 4). We do note that reports of no RI (none) occurred in association with all observed cell abundances, reflecting the importance of wind direction and/or speed on RI risk as discussed below.

### 4.2 Wind Direction and Speed as Modulators of RI Risk Due To Red Tide

If *K. brevis* cells are present, the model assumes that there is a higher risk of RI if winds are blowing onshore and secondarily that there will be higher risk of RI with higher onshore winds. However, contrary to our hypothesis, when *K. brevis* cells were present there were more instances of slight RI reported at Venice Beach, FL during offshore winds than onshore (Figure 5b), which was also true for our analysis at all beaches along the southwest Florida coast (Figure S2b in Supporting Information S1). We also found more instances of moderate RI when winds were offshore (Figure S2c in Supporting Information S1); however, there were substantially more instances of intense RI when winds were onshore (Figure S2d in Supporting Information S1). This suggests that wind direction may play a role in modulating risk of RI at the extremes—for example, when *K. brevis* cells were present at high concentrations, onshore winds likely led to increased risk of RI (Figure S2d in Supporting Information S1). For occurrences of slight and moderate RI, wind direction did not appear to have a substantial effect on modeled RI risk (Figure S2b in Supporting Information S1).

Wind speed also modulated the risk of RI, especially at higher cell abundances when winds were onshore and *K. brevis* cells were present (Figure 6). Supporting model assumptions, when there were high cell abundances (>1,000,000 cells L^−1^) there was a statistically higher risk of RI with increasing wind speeds (Figure 6). In addition, there was also a statistically higher risk of slight RI, as compared to none, at higher wind speeds when cells were present at the low category (>10,000–100,000 cells L^−1^); however, this assumption did not hold for very low or medium *K. brevis* cell abundance categories (Figure 6). This indicates that wind speed may only be a significant factor in predicting beach‐level RI when cell abundances are high and further supports evidence of the low predictive ability of the model at low and medium cell abundances.

### 4.3 Model Limitations

The RI model proved to be 88% accurate at predicting RI risk when *K. brevis* was present along the southwest Florida coast. However, even when *K. brevis* presence was confirmed along the southwest Florida coast, this accuracy was largely driven by the 87% accuracy for predicting a very low risk of RI (Table 2). Despite the low prevalence of intense RI, the model was 61% accurate at predicting these high risk events (Table 2), thus providing important warning to beachgoers of intense RI risk. We do note that the model greatly overpredicted the occurrence of high/intense RI (Bias = 4.17; Table 2). We argue that there can be specific advantages in biasing toward more warnings, particularly at the highest risk level. Failure to appropriately warn potential beach‐goers of high risk events (e.g., FN) can negatively impact human health or lead to a lack of confidence in the forecast as someone may go to the beach and experience intense RI. In contrast, someone who avoids the beach during a high risk forecast when no impacts were actually observed (e.g., FP), results in no actual harm to the individual, although it may reduce use of beach‐side businesses. Conversely, the model had very low user's accuracy at the intermediate observed RI risk (low, moderate), which as discussed above, may be due to the limited influence of onshore versus offshore winds and wind speed at the intermediate RI risk. Additional work is needed to improve model performance at these intermediate levels, which is particularly important for beach‐goers with underlying respiratory conditions who may be disproportionately impacted even when RI risk and impacts are relatively mild for the general population (Fleming et al., 2011; Kirkpatrick et al., 2006).

There were also additional model assumptions that may be obscuring the accuracy of the RI model. First, the model assumes that only cells immediately adjacent to the beach are the source of red tide induced RI observed by BCRS RI reports. However, Kirkpatrick et al. (2010) showed that during severe bloom events, instances of reported RI can extend over 1 km inland. Thus, a dense bloom may occur a short distance off the beach, potentially resulting in RI even if beach‐level sampling within the surf or just offshore shows low *K. brevis* cell abundances (Figure S3 in Supporting Information S1). *K. brevis* blooms, and associated sampling efforts, are also inherently patchy over space and time; thus, even routine sampling programs may potentially miss a dense bloom patch adjacent to the sampling location. Methods for collecting additional spatial data along and just offshore (up to 1 km) would help alleviate this problem, especially during known red tide bloom events. Satellite and other remote sensing data may also help to increase sampling spatial resolution, although current methods for daily satellite remote sensing monitoring are limited to 300 m pixels (Wynne et al., 2021), which limits cell detection close to shore. In addition, the limit of detection for remote sensing pixels has been estimated at 50,000 cells L^−1^, which is greater than *K. brevis* cell abundances that may cause RI in those with underlying health conditions (Stumpf et al., 2009). New technologies with higher spatial and temporal resolution, including new satellite sensors (e.g., Sentinel‐2, NASA's PACE) or unmanned aerial systems, may provide additional spatial sampling support.

Finally, in the current model, we do not account for RI induced when *K. brevis* is present in the bays and inlets of southwest Florida. *K. brevis* does regularly enter inlets and bays behind barrier islands, especially during particularly intense bloom events (FWRI, 2024). As barrier islands are typically less than 1 km wide, a bayside bloom coupled with offshore winds could induce RI at beachside locations, which is not accounted for in the current model design.

### 4.4 BCRS Limitations

RI reports collected by BCRS rely on a full‐time lifeguard corps and trained beach volunteers, many of whom have worked for years along the coast (Kirkpatrick et al., 2008). The rigorous training and consistent personnel provide a level of consistency among BCRS observations; however, error and bias are possible and may be inherent in the BCRS‐based validation data set used here. For example, it is relatively straightforward to categorize RI into the two extremes (i.e., none vs. intense) but may be more difficult to assess intermediate categories (i.e., slight vs. moderate), especially if there are confounding factors such as the number of beach goers or time of day. In addition, the problem of locating threshold boundaries is well recognized, and for continuous data, can be handled using methods such as the receiver operator characteristics (ROC; Metz, 2006); additional work is likely needed to better separate the different categories of RI. In addition, while the number of coughs are likely a relatively robust metric of red tide induced RI at beaches, there could be additional factors that lead to coughing including other illnesses, seawater ingestion, or presence of other aerosolized irritants (e.g., smoke, smog, etc.).

### 4.5 Incorporating Multiple Pathways of Exposure in RI Risk Assessment

Finally, the model assumes RI risk is only due to exposure via aerosolized brevetoxins on the beach (e.g., walking or sitting) and does not account for exposure to brevetoxins when beachgoers may be swimming, wading, or engaging in other behavior which would lead to brevetoxin exposure regardless of wind direction. While direct water exposure may cause respiratory symptoms that would be, accurately, identified by BCRS, they are not currently accounted for in the model. Also, increased reports of RI during offshore winds may be a result of more pleasant conditions at the beach, and therefore more swimming activity and brevetoxin exposure through direct water contact. A measure of both aerosolized and direct (e.g., swimming, water) brevetoxin exposure is necessary to capture impacts of RI due to *K. brevis* from multiple exposure methods. In addition, future work on impacts related to brevetoxin exposure should consider beachgoer density and behavior associated with wind and aerosol conditions.

## 5 Conclusions

Overall, the NOAA RI model performed well throughout the study period (2006–2022), especially at predicting very low/none and high/intense risk of RI due to *K. brevis*, and provides critical information to beach‐goers on potential severity and impacts of red tide induced RI at individual beaches along the southwest Florida coast. However, the model is less robust at predicting low/slight and moderate instances of RI, with more nuanced relationships between RI risk and wind direction/speed than originally hypothesized. For example, the potential for a low/slight risk of RI may occur more frequently and under a broader range of conditions than originally built into the model. Additional work is needed to better constrain the environmental conditions leading to brevetoxin exposure, including toxin aerosolization mechanisms, and RI symptoms at these low and moderate levels. In addition, an inherent limitation of the current model and the BCRS validation data set is the implicit link between *K. brevis* cell presence in the water to symptoms (e.g., coughing) at the beach, without specifically measuring brevetoxin exposure or aerosolization. Only a few studies have measured aerosol presence to date (Pierce et al., 2005) and there is a clear need for robust methodologies to determine aerosolized brevetoxins over a short time period (e.g., hours) and under a range of wind conditions (wind speed and direction). In addition, high spatial resolution *K. brevis* monitoring would help to better constrain inherent bloom patchiness and potential exposure. Overall, predictive models, such as the RI risk model presented here, are critical tools for providing advanced warning and relevant information to the public on the human health risk associated with red tide (Hoagland et al., 2020). Additional work to improve the accuracy, resolution, and predictive capabilities of the RI model, as highlighted above, will continue to advance the use of these tools for minimizing the negative impacts of red tide on coastal communities in southwest Florida.

## Disclaimer

The scientific results and conclusions, as well as any views or opinions expressed herein, are those of the author(s) and do not necessarily reflect those of NOAA or the Department of Commerce.

## Conflict of Interest

KMC was employed by CSS Inc. The remaining authors declare that the research was conducted in the absence of any commercial or financial relationships that could be construed as a potential conflict of interest.

## Supporting information

Supporting Information S1

## Data Availability

*Karenia brevis* cell abundance data from FWRI and partners can be obtained via the Harmful Algal BloomS Observing System (NOAA National Centers for Environmental Information, 2014). Cell abundance categories from Pinellas County, the SCCF, and HABscope can be found in the archived RI forecasts for southwest Florida with NOAA's National Centers for Environmental Information (NOAA National Ocean Service, 2017). Wind speed and direction data can be found with NOAA's National Buoy Data Center (NDBC), including NOAA National Ocean Service (2025a, 2025b) and NOAA National Buoy Data Center, (2025). Additionally, code used to generate model hindcasts (2006–2022) as well as code for statistical analysis and visualizations are archived via Zenodo (Hounshell & Sellers, 2025).
