# Supplementary material for: Predicting Karenia brevis Induced Respiratory Irritation at Individual Southwest Florida Beaches Using Cell Abundances Plus Wind Direction and Speed
Source: Geohealth. 2026 Apr 9;10(4):e2025GH001664. doi: 10.1029/2025GH001664 (PMC13062942; doi:10.1029/2025GH001664)
Supplement: Supplementary file 1 — Supporting Information S1 [file GH2-10-e2025GH001664-s001.pdf]

GeoHealth

Supporting Information for

Predicting *Karenia brevis* induced respiratory irritation at individual southwest Florida beaches using cell abundances plus wind direction and speed

K. M. Collins[1], A. G. Hounshell[2*], B. Kirkpatrick[3], A. Cook[4], K. A. Hubbard[5], M. C. Tomlinson[6], R. P. Stumpf[6]

[1] CSS Inc. at National Oceanic and Atmospheric Administration, National Centers for Coastal Ocean Science, Silver Spring, Maryland, USA

[2] National Oceanic and Atmospheric Administration, National Centers for Coastal Ocean Science, Beaufort, North Carolina, USA

[3] Gulf of America Coastal Ocean Observing System, Texas A&M University, College Stations, Texas, USA

4 Mote Marine Laboratory, Sarasota, Florida, USA

[5] Florida Fish and Wildlife Commission, Fish and Wildlife Research Institute, St. Petersburg, Florida, USA

[6] National Oceanic and Atmospheric Administration, National Centers for Coastal Ocean Science, Silver Spring, Maryland, USA

[*] Corresponding author: Alexandria G. Hounshell, alexandria.hounshell@noaa.gov

Contents of this file

Figures S1 to S3
Tables S1 to S2

Table S1. Multi-category contingency table showing instances of matched cell abundance category and observed BCRS respiratory irritation from 2006-2022.

		BCRS Respiratory Irritation Category				
		**None**	**Slight**	**Moderate**	**Intense**	**Total**
**Cell Abundance Category**	**Background**	7,978	167	39	7	8,191
	**Very Low**	651	90	21	1	763
	**Low**	832	195	46	3	1,076
	**Medium**	586	343	87	13	1,029
	**High**	175	176	171	50	572
	**Total**	10,222	971	364	74	11,631

Table S2. Kruskal-Wallis with post-hoc Cliff's delta results to identify statistical differences among BCRS respiratory irritation risk by observed cell abundance category and wind speed. For Kurskal-Wallis a p-value of 0.05 was used to assess if groups were statistically different. Post-hoc Cliff's delta was calculated for the Low and High cell count categories. The following categories were used to designate the effect size: Negligible<|0.15|; Small |0.15|-|0.33|; Median |0.33|-|0.47|; Large>|0.47| (Meissel and Yao, 2024).

**Cell Count Category**	**Kruskal-Wallis *p***		
Very Low	0.13		
Low	0.004		
**Post-hoc Cliff's Delta**			
BCRS RI	**None**	**Slight**	**Moderate**
Slight	-0.26	-	
Moderate	-0.20	0.12	-
Intense	-0.07	0.13	0.15
Medium	0.06		
High	<0.005		
**Post-hoc Cliff's Delta**			
**BCRS RI**	**None**	**Slight**	**Moderate**
Slight	-0.38	-	-
Moderate	-0.41	-0.04	-
Intense	-0.67	-0.25	-0.20

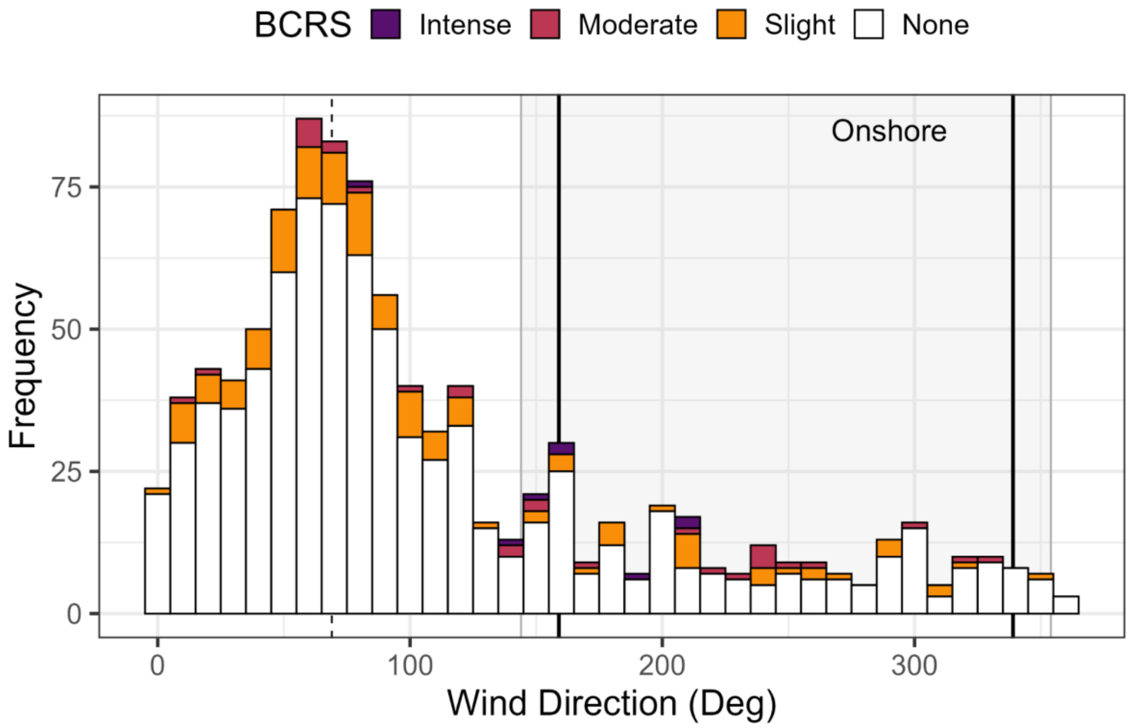

Figure S1. Frequency plots of wind direction (degrees) of the BCRS respiratory irritation categories (None, Slight, Moderate, Intense) for Venice Beach, Florida from 2006-2022 (n=956). Dashed, vertical lines correspond to the 90° shoreline angle (69°). Solid, vertical lines indicate the 90° shoreline angle±90° (159° and 339°, respectively). The light grey box indicates the wind direction considered offshore winds (shoreline angle, 144° and 354°, respectively).

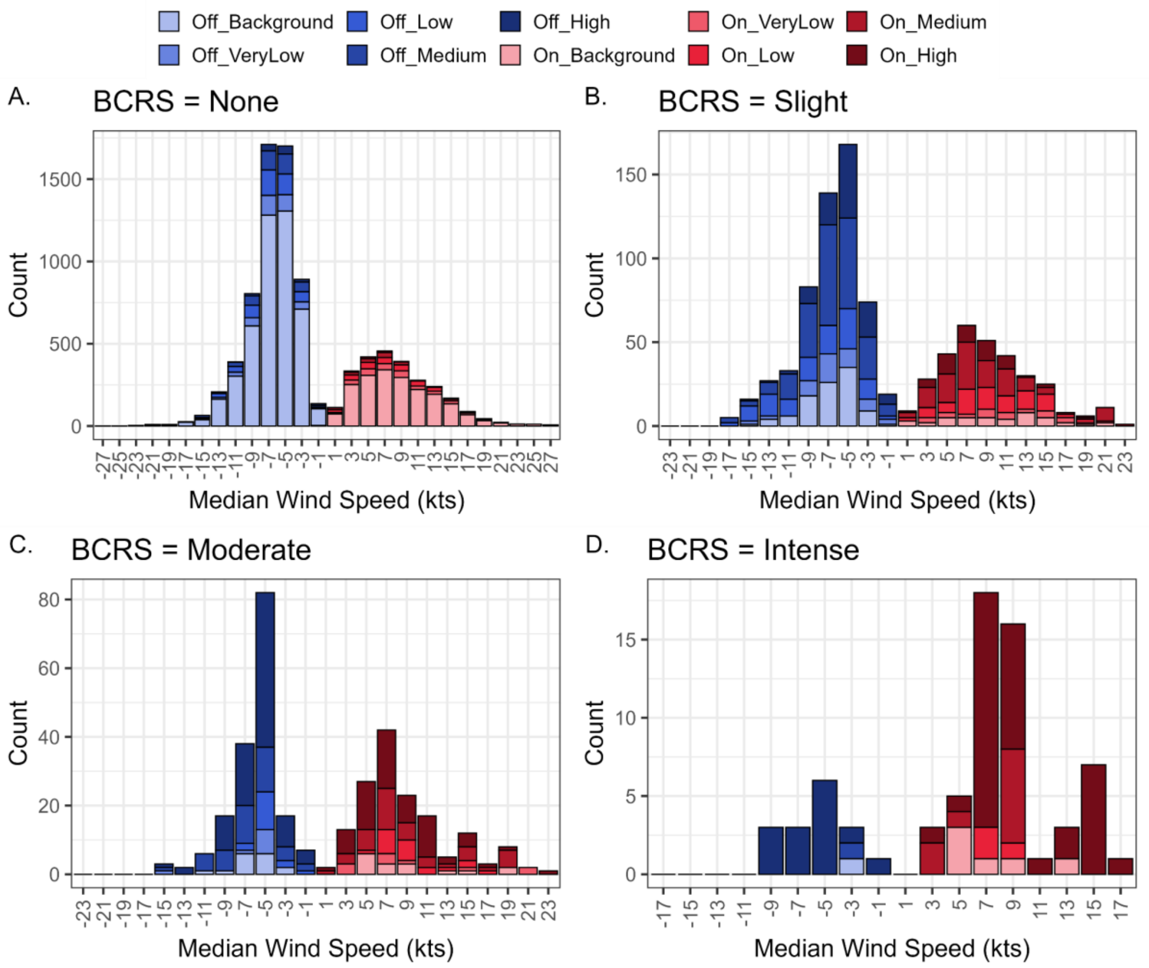

Figure S2. Histograms showing the distribution of BCRS reported RI categorized by wind speed (knots, kts) for A. None, B. Slight, C. Moderate, and D. Intense RI across all locations from 2006-2022. Blue corresponds to offshore winds, while red corresponds to onshore winds. Color variation corresponds to cell abundance category from background to high cell concentrations. Note the changing y- and x-axes.

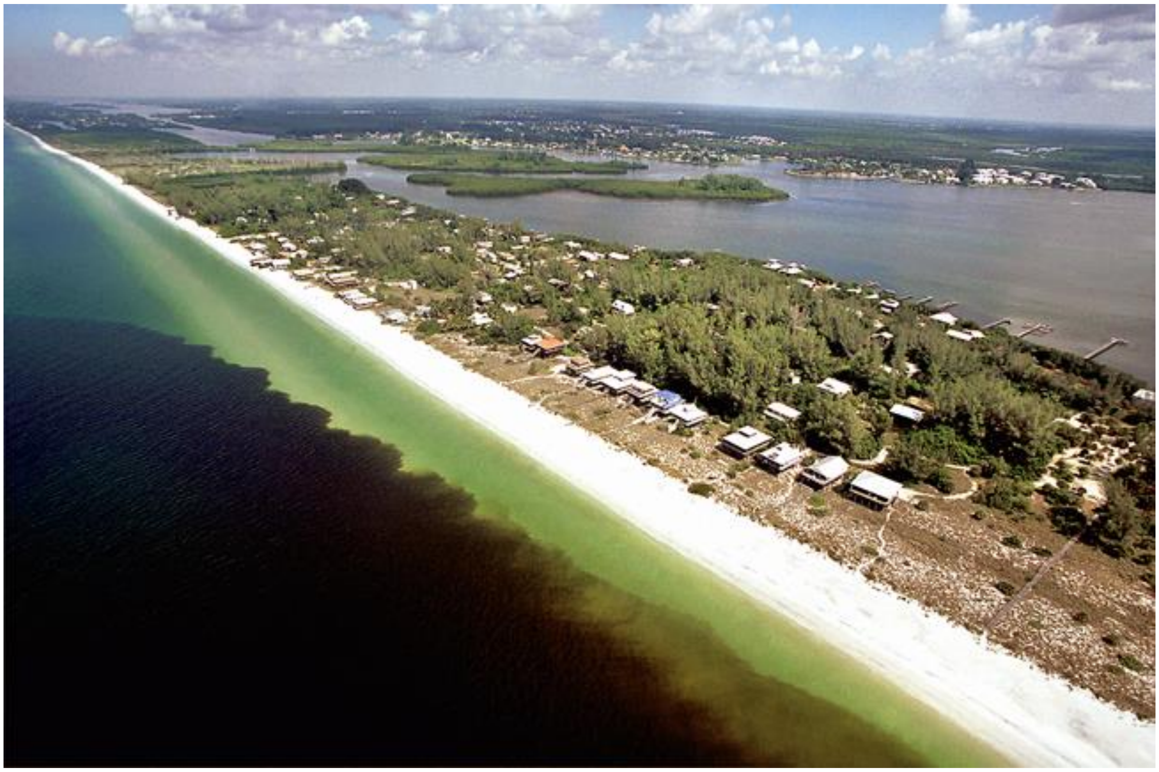

Figure S3. Aerial image of red tide (*Karenia brevis*) along the southwest Florida coast, showing the patchiness of blooms that can aggregate just offshore and potentially cause RI from aerosolized brevetoxins. Photo Credit: Paul Schmidt, Charlotte Sun Herald.
